# Effects of long-term consumption of two plant-based dietary supplements on cardiovascular health and low-grade inflammation in middle-aged and elderly people: study protocol for a randomised, controlled trial

**DOI:** 10.1186/s41043-023-00434-x

**Published:** 2023-09-19

**Authors:** Melina Tsiountsioura, Gerhard Cvirn, Lisa Meixner-Goetz, Tobias Ziegler, Manfred Lamprecht

**Affiliations:** 1 Juice Plus+ Science Institute, 140 Crescent Drive, Collierville, TN 38017 USA; 2 Division of Medicinal Chemistry, Otto Loewi Research Center, Medical University of Graz, Neue Stiftingtalstrasse 6/III, 8010 Graz, Austria; 3 Green Beat - Institute of Nutrient Research and Sport Nutrition, Nernstgasse 1, 8010 Graz, Austria

**Keywords:** Ageing, CVD prevention, Low-grade inflammation, Fruits, Vegetables, Omega-3 fatty acids, Dietary supplements

## Abstract

**Background:**

Ageing is a process characterised by chronic low-grade inflammation and oxidative stress which could lead to increased prevalence of both physical and mental age-related chronic conditions. A healthy balanced diet, rich in fruit and vegetables as well as omega-3 polyunsaturated fatty acids (n3 PUFA), could reduce oxidative stress and improve markers of low-grade inflammation. Nonetheless, considering that a large part of the population struggles to meet current guidelines on fruit and vegetable and n3 PUFA recommendations, fruit and vegetable concentrate supplements and mixed omega fatty acid supplements could be an effective strategy to bridge the gap between actual and recommended intakes.

**Methods:**

In this randomised, controlled, open-labelled, parallel-grouped clinical trial, 112 participants will be allocated to one of four arms (*n* = 28 on each arm): an encapsulated juice powder concentrate, a plant-based omega fatty acid supplement, both or a control group. We aim to investigate whether long-term separate or combined ingestion of the two can affect biomarkers of cardiovascular health, low-grade inflammation and indicators of ageing, including cognitive function, in middle-aged and elderly people. We will additionally explore the effect of the different supplementations on plasma levels of vitamins, carotenoids and fatty acids. Intervention will last 2 years and participants will be assessed at baseline and at follow-up visits at 6, 12, 18 and 24 months.

**Discussion:**

This study will provide evidence whether long-term, plant-based dietary supplementation can support cardiovascular health, anti-inflammatory processes, immunity and nutritional status in ageing.

*Trial registration* This trial was registered at ClinicalTrials.gov (NCT04763291) on February 21, 2021.

## Background

The global elderly population has been growing exponentially in the last few years, and according to the World Health Organization, it is estimated that it will nearly double between 2015 and 2050 [1]. This results in an increased prevalence of age-related chronic conditions, including atherosclerosis and cardiovascular diseases (CVD), insulin resistance and type 2 diabetes, cognitive decline and diminished mental health [2]. Many of these conditions are interrelated. For example, ageing in blood vessels could not only lead to the development of vascular diseases, but could also affect the central nervous system, where cerebral vascular ageing could lead to cognitive decline [3].

Dietary intake is a modifiable risk factor that could affect the incidence and severity of age-related functional decline and prevalence of the aforementioned age-related diseases. Evidence from previous epidemiological studies supports a strong relationship between fruit and vegetable consumption and reduced cardiac risk [4, 5]. This could be mediated via improvements in blood pressure, platelet function and vascular reactivity; yet, the mechanisms of protection are not clear. Increased fruit and vegetable consumption has also been associated with reduced inflammation and enhanced immune cell profile, according to a recent systematic review and meta-analysis [6]. Some epidemiological studies have reported that fruit and vegetable intake is inversely associated with proinflammatory biomarkers, such as C-reactive protein (CRP) [7] and tumour necrosis factor alpha (TNF-α) [8]. In addition, inflammation is closely related to oxidative stress, and since one process can easily induce the other, they are often found together in many pathological conditions [9]. It is known that certain vitamins and polyphenols found in fruits and vegetables protect against oxidative stress by scavenging free radicals [10, 11]. Vitamin C, for instance, reduces cellular oxidative stress and improves endothelial function [10]. Vitamin E, another potent antioxidant, has been shown to decrease lipid peroxidation and improves endothelial function by increasing plasma nitric oxide [11]. Beneficial effects have also been observed for higher fruit and vegetable intake—and consequently antioxidants—and cognitive health, including age-related cognitive impairment [12]. This is achieved by the antioxidant and anti-inflammatory activities of these compounds and their ability to improve cellular signalling and neuronal communication [13].

Previous studies have also demonstrated the importance of long-chain omega-3 fatty acids on humans’ health and their positive effects on the cardiovascular system and blood lipids regulation [14, 15]. Long-chain omega-3 polyunsaturated fatty acids (n3 PUFA) are also considered to have anti-inflammatory effects [2], and increased long-chain n3 PUFA intake has been associated with reduced circulating levels of pro-inflammatory cytokines in the elderly [16]. The n3 PUFA eicosapentaenoic acid (EPA) and docosahexaenoic acid (DHA) are essential fatty acids that humans are not able to synthesise, and hence have to be supplied through the diet [15].

Dietary patterns with adequate intake of fruit and vegetables, micronutrients and n3 PUFA can reduce oxidative stress and improve markers of low-grade inflammation, cardiovascular risk profile and cognitive function [17–22]. According to the Austrian nutrition report and the Austrian Health survey, Austrian adults consume on average two out of the five recommended portions of fruit and vegetables per day [23] and only half of the population consumes one portion of fish per week, with the remaining half consuming lower amounts [24]. Similar patterns are observed in other western countries [25, 26]. Considering that many people’s fruit and vegetable and n3 PUFA intakes are below recommendations, supplementation with fruit and vegetable concentrates and mixed omega fatty acid supplements could potentially exert beneficial effects on their health. Previous short-term studies have shown that supplementation with fruit and vegetable concentrates is able to reduce low-grade inflammation and oxidative stress in different cohorts of people [27, 28]. Little is known though about the effect of such supplements over the long term. The aim of this study is to investigate whether long-term separate ingestions of an encapsulated juice powder concentrate or a plant-based omega fatty acid supplement, or a combined ingestion of the two, can affect biomarkers of cardiovascular health, low-grade inflammation and indicators of ageing in middle-aged and elderly people.

## Methods

### Study design

This is a randomised, controlled, open-label, parallel-grouped clinical trial. Enrolled participants will be stratified by gender and randomly allocated to one of the following arms:

- Control group (no intervention)
- Fruit, Vegetable and Berry (FVB) group, where participants have to ingest an encapsulated fruit, vegetable and berry juice powder concentrate.
- Omega group, where participants have to ingest an encapsulated plant-based fatty acid supplement.
- Fruit, Vegetable, Berry and Omega (FVBO) group, where participants have to ingest the fruit, vegetable and berry supplement, together with the plant-based fatty acid supplement.

Randomisation will be performed by a true random number service that generates randomness via atmospheric noise (‘RANDOM.ORG’, Randomness and Integrity Services Ltd., Dublin, IE), by an independent statistician not involved in the recruitment process or analysis of data. In order to ensure a balanced sample size across groups over time, we will stratify by gender and perform block randomisation with a block size of 4. Allocation concealment will be ensured, as the independent statistician will only release the randomisation code to the study team, after a participant is fully enrolled in the study.

The intervention will last 24 months and participants in all groups are advised to continue their habitual diet and lifestyle, throughout the whole duration of the study.

### Recruitment and trial setting

Participants will be recruited from the Graz area, Austria, either by word of mouth or by replying to advertising material such as leaflets placed in public places including gyms, medical practices, pharmacies, seniors’ associations and clubs or via social media announcements.

Screening visits will take place at the Greenbeat Institute of Nutrient Research and Sports Nutrition. All remaining visits will take place at the Otto Loewi Research Center, Division of Medicinal Chemistry of the Medical University of Graz, Austria.

### Participants

#### Inclusion criteria

- Males and females
- Age: 55–80 years
- Post-menopausal
- Non-smokers
- Body mass index (BMI): 18.5–40 kg/ m^2^
- Fruit and vegetable intake: ≤ 4 servings/ day
- Adherence to a 6-week washout period, for dietary supplements not ingested for specific medical conditions.

#### Exclusion criteria

- Age: < 55 and > 80 years
- Smokers (or ex-smokers who quit smoking < 3 years ago)
- Aversion to stop the intake of multivitamins, multiminerals or omega fatty acid supplements
- Subjects with histamine intolerance
- Hypertension, starting with grade 2 according to the classification of the European Society of Hypertension (systolic blood pressure > 160 mmHg; diastolic blood pressure > 100 mmHg)
- All medication taken for less than 3 months or with changes on the dosage over the last 3 months
- Medication for any of the conditions listed below
- Clinically relevant infectious diseases
- All forms of dementia and Alzheimer’s disease
- Diabetes mellitus type I and type II
- Autoimmune diseases
- Stents and cardiovascular diseases
- Cancer patients
- Pregnancy
- Significant lifestyle changes, e.g. changes in diet and physical activity profile

### Interventions

#### Encapsulated fruit, vegetable and berry juice powder concentrate

The encapsulated fruit, vegetable and berry juice powder concentrate (Juice Plus + ®capsules) is a vitamin- and phytonutrient-rich supplement, derived from 36 dried fruits, vegetables and berries. Participants in the FVB and FVBO groups will be asked to ingest 6 capsules per day, which provide 2910 µg β-carotene, 158mg vitamin C, 18.2mg α-tocopherol equivalents (α-TE, vitamin E), 316 μg folic acid, 6.1mg lutein, 1mg lycopene, > 100 different phenolic compounds and approximately 600mg of polyphenols [29]. Table 1 provides detailed information on the recommended daily allowance (RDA)/ adequate intake (AI)—for available nutrients—contained in the FVB capsules as well as the % RDA/ AI covered by the ingestion of 6 capsules per day, based on the nutrient reference values for the DACH region (collaboration between the German, Austrian and Swiss nutrition societies) [30].

**Table 1:** Nutrient reference values and % coverage offered by FVB and Omega capsules

Nutrient	Gender and age range	DACH/EFSA RDA/ AI	FVB (6 capsules)	DACH % RDA/ AI covered by FVB capsules (%)	Omega (2 capsules)	EFSA % AI covered by Omega capsules
β-carotene*	M; 51–65 years	10,200 μg	2910 μg	28.5	–	–
	M; > 65 years	9600 μg	2910 μg	30.3	–	–
	F; > 51 years	8400 μg	2910 μg	34.6	–	–
Vitamin C*	M	110 mg	158 mg	143.6	–	–
	F	95 mg	158 mg	166.3	–	–
α-tocopherol**	M; 51–65 years	13 mg	18.2 mg	140	–	–
	M; > 65 years	12 mg	18.2 mg	151.2	–	–
	F; 51–65 years	12 mg	18.2 mg	151.2	–	–
	F; > 65 years	11 mg	18.2 mg	165.5	–	–
Folic acid*	M & F	180 μg	316 μg	175.6	–	–
EPA + DHA**	M & F	250	–	–	275	110%

*AI* adequate intake, *DACH* Germany, Austria, Switzerland, *DHA* docosahexaenoic acid, *EFSA* European Food Safety Authority, *EPA* eicosapentaenoic acid, *F* female, *FVB* fruit, vegetable and berry, *M* male, *RDA* recommended dietary allowance

*Presented as recommended dietary allowance (RDA)

**Presented as adequate intake (AI)

#### Encapsulated plant-based fatty acid supplement

The encapsulated fatty acid supplement (Juice Plus + ®Omega Blend) contains plant-based omega fatty acids from algae (*Schizochytrium sp.*) and a variety of seed oils. Participants in the Omega and FVBO groups will be asked to ingest two capsules per day, which provide 375mg of n3 fatty acids [175 mg of DHA, 100mg of EPA, 55mg of docosapentaenoic acid (DPA) and 35mg of α-linolenic acid (ALA)], 250mg of n5 and n7 fatty acids, and 250 mg of n6 and n9 fatty acids. Table 1 provides information on the adequate intake (AI)—for available nutrients—contained in the Omega capsules as well as the % AI covered by the ingestion of 2 capsules per day, based on the nutrient reference values from the European Food Safety Authority (EFSA) [31].

Based on previous experience and in order to increase tolerability, participants are recommended to take capsules with a meal, preferably in the morning and evening. Previous studies have tested the safety of the supplements used and reported no effects on liver, kidney and thyroid function [32, 33].

### Study outcomes

The co-primary outcomes will be changes on markers of cardiovascular health and low-grade inflammation following 24 months of supplementation. Secondary outcomes include changes on selected indicators of ageing, upper respiratory tract symptoms and quality of life. Finally, tertiary outcomes include plasma concentration changes of vitamins, carotenoids and fatty acids.

A detailed summary of all parameters to be assessed is given in Table 2.

**Table 2:** Detailed summary of primary, secondary and tertiary outcome parameters

Primary outcomes	Low-grade inflammation
Cardiovascular health	
Total cholesterol, LDL, HDL, ApoA1, TG, oxLDL, homocysteine, omega-3 Index, platelet aggregation, TEM, coagulation (PT, PTT), glucose, insulin, HOMA-IR, HbA1c, MDA, CP, redox state of albumin	TNF-α, sTNFR1 and sTNFR2, CCL5, IL-1β, hsCRP, CCL2, OPG, IL-5, IL-8

Secondary outcomes	Upper respiratory tract symptoms	Quality of life
Selected indicators of ageing		
uc-osteocalcin, dp-ucMGP, mtDNA-CN, BDNF, NGF, CFD index	WURSS-21	SF-36

Tertiary outcomes	Fatty acids
Vitamins and carotenoids	
Retinol, vitamins C, D, E (α-tocopherol and γ- tocopherol), K1, K2 (MK-7), α-carotene, β-carotene, lutein, zeaxanthin, lycopene, β-cryptoxanthin	DHA, DPA, EPA, AA, ALA, linoleic acid, oleic acid, palmitoleic acid, stearic acid

*AA* arachidonic acid, *ALA* alpha-linolenic acid, *ApoA1* apolipoprotein A1, *BDNF* brain-derived neurotropic factor, *CCL2* chemokine (C–C motif) ligand 2, *CCL5* chemokine (C–C motif) ligand 5, *CFD index* cognitive functions dementia index, *CP* carbonyl proteins, *DHA* docosahexaenoic acid, *DPA* docosapentaenoic acid, *dp-ucMGP* dephosphorylated-uncarboxylated Matrix GLA protein, *EPA* eicosapentaenoic acid, *HbA1c* haemoglobin A1c, *HDL* high-density lipoprotein, *HOMA-IR* homeostatic model assessment of insulin resistance, *hsCRP* high-sensitivity C-reactive protein, *IL-1β* interleukin 1-beta, *IL-5* Interleukin-5, *IL-8* interleukin-8, *LDL* low-density lipoprotein, *MDA* malondialdehyde, *mtDNA-CN* mitochondrial DNA copy number, *NGF* nerve growth factor, *OPG* osteoprotegerin, *oxLDL* oxidised low-density lipoprotein, *PT* prothrombin time, *PTT* partial thromboplastin time, *SF-36* 36-Item Short Form Health Survey, *sTNFR1* soluble tumour necrosis factor receptor 1, *sTNFR2* soluble tumour necrosis factor receptor 2, *TEM* thrombelastometry, *TG* triglycerides, *TNF-α* tumour necrosis factor alpha, *uc-osteocalcin* undercarboxylated osteocalcin, *WURSS-21* Wisconsin upper respiratory symptom survey

### Study visits and procedures

#### Screening visit

Subjects expressing interest to participate in the study will be invited to a screening visit. On the screening visit, a research team member will go through the participant information sheet and explain all study procedures to potential participants. Participants will have the opportunity to ask any questions they might have. If they are still interested to participate, they will be asked to sign an informed consent form, prior to any measurements being taken.

During the screening visit, participants’ medical history will be assessed and measurements of height, weight, BMI and blood pressure (BP) will be taken. Participants meeting the inclusion criteria will be enrolled in the study and an appointment for the baseline visit will be arranged. A minimum of 6-week washout period, to eliminate any interfering nutritional supplements and dietetic products, will be allowed between the screening and baseline visit. Also, in order to avoid a potential effect of physical activity and changed nutritional habits on the measured blood markers, participants will be asked to abstain from any form of strenuous physical activity and complete an estimated food diary, 4 days prior to the baseline visit. Detailed information on how to complete the food diary will be provided during the screening visit.

#### Baseline visit

During the baseline visit, the study physician will assess participants’ history once again to ensure that they still fit the inclusion criteria of the study.

Height, weight, BMI and blood pressure will be measured. Participants will be required to fast overnight and fasting blood samples will be collected by venepuncture, to measure biomarkers of cardiovascular health, low-grade inflammation, indicators of ageing, circulating micronutrients and fatty acids.

Following blood drawing, participants will be offered a snack and then asked to complete the cognitive function test (CFD index). Subsequently, they will complete the Short Form survey (SF-36) and the Perceived Stress Scale (PSS-10) questionnaires, which assess quality of life and stress profile respectively. In addition, participants’ physical activity will be assessed via completion of the International Physical Activity Questionnaire—Short Form (IPAQ-SF).

As part of safety routine, we will perform blood analysis including clinical chemistry [i.e. key biomarkers of liver (aspartate transaminase, alkaline phosphatase, gamma-glutamyl transferase) and kidney function (urea, uric acid, creatinine, glomerular filtration rate)], blood proteins, haemogram and coagulation markers. We will also collect the food diary, which was provided to participants during the screening visit, to assess their nutrition and ensure they do not exceed the fruit and vegetable intake, outlined in the inclusion/ exclusion criteria.

At the end of the baseline visit, participants will be randomly allocated to one of the four groups. Participants in the control group will continue with their habitual diet and lifestyle. Those allocated to the three whole food supplement groups, will have to continue their habitual diet and lifestyle, and will also be provided with the corresponding supplements they will have to ingest. Participants will be asked to avoid disposing of any unused capsules and return any leftovers in the next follow-up visit. In addition, all participants will receive a copy of the Wisconsin Upper Respiratory tract Symptoms Survey (WURSS-21) and the Gastro-Intestinal Symptoms Survey-16 (GISS-16) and will be asked to fill these in every time they experience upper respiratory tract or gastrointestinal symptoms, during the duration of the intervention. They will also be reminded that over the following two years they will have to maintain their habitual diet which must correspond to the nutritional inclusion and exclusion criteria.

#### Follow-up visits

Follow-up visits at 12 and 24 months will be identical to the baseline visit. Follow-up visits at 6 and 18 months will be the same with the baseline visit, with the only exception that the cognitive function test and mtDNA-CN will not be performed. This is due to the fact that we estimate that more than 6-month time would be required to detect any potential changes in these parameters [34].

Participants will be provided with a copy of the food diary they had completed prior to the baseline visit and will be asked to replicate their diet, 4 days before each follow-up visit, to avoid any potential effect of changed nutritional habits on the measured blood markers. They will also be reminded to avoid any strenuous physical activity during this time. At every follow-up visit, we will collect any WURSS-21 and GISS-16 questionnaires completed during the previous 6 months, from participants that experienced respiratory tract symptoms or gastrointestinal discomfort. We will also collect any leftover capsules from the previous 6 months, to monitor intervention compliance. Participants will be contacted via telephone in between visits with a cadence of 3 months, by a member of the research team, to keep them motivated and ensure maximum retention to the trial.

**Fig. 1:**
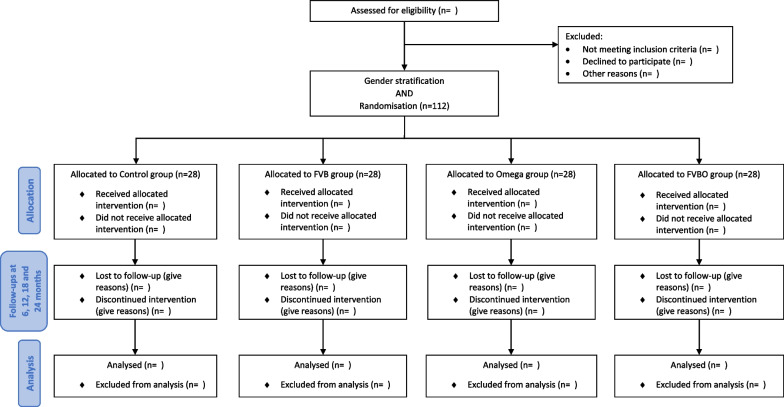
CONSORT flow diagram

**Fig. 2:**
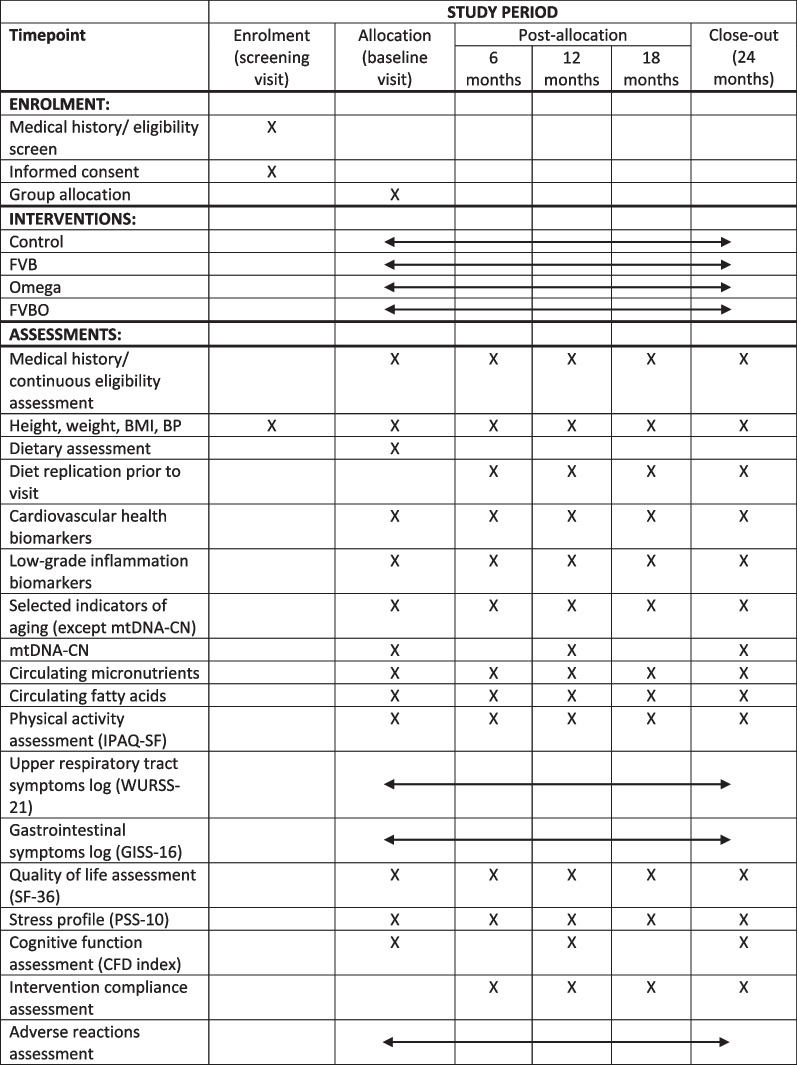
SPIRIT figure showing the schedule of interventions and assessments

Figure 1 summarises participants’ recruitment and follow-up plan, according to the Consolidated Standards of Reporting Trials (CONSORT) 2010 statement. Figure 2, provides an overview of the schedule of interventions and all study procedures performed at each study visit, according to the Standard Protocol Items: Recommendations for Interventional Trials (SPIRIT) guidelines [35].

#### Adverse events assessment and reporting

An adverse events reporting system is in place for this study. Any adverse events will be collected after a participant has provided consent and enrolled in the study. The study physician will continuously assess participants’ medical history throughout the study and will refer participants to a specialist expert, should this be necessary. In case the health status of a participant changes during the study or in case of any other concern, the study physician will assess the continuation or withdrawal of the participant from the study.

#### Ancillary care

Participants enrolled in this study are covered by liability insurance provided by the supplementation company and is valid for the entire duration of the study.

### Analytical assessments and measurement tools

#### Height, weight, BMI and blood pressure

Height will be measured to the nearest 0.1 cm using a stadiometer (Seca 217) and weight to the nearest 0.1 kg using calibrated mass scales (Seca robusta 813). BMI will be calculated by dividing weight in kg by the square of height in m. Two blood pressure readings on each arm, using an automated blood pressure monitor (Boso medicus x), will be recorded while the participant is in a seated position.

#### Blood markers

A total of 47 ml from each participant will be collected by venepuncture by the study physician at each visit for the analysis of blood biomarkers of cardiovascular health, low-grade inflammation, selected indicators of ageing, vitamins, carotenoids and fatty acids. Aggregation and thrombelastometry parameters will be immediately measured in fresh whole blood. 1mL of whole blood will be stored at −80°C for future analysis of mtDNA. Blood samples for the clinical chemistry analyses will be transported immediately after collection to another collaborating laboratory for analysis. Remaining blood will be centrifuged at 3000 rpm for 10 min to get the plasma. The supernatant will be frozen at −80°C and kept at the study site for further analysis.

Blood lipids including total cholesterol, LDL, HDL, triglycerides and ApoA1, as well as homocysteine, hsCRP and vitamin D will be measured by the standard equipment Architect ci8200 (Abbott Diagnostics, IL, USA). Glucose and HbA1c will be measured photometrically using the Alinity system (Abbott, IL, USA) and insulin by the fully automated chemiluminescence analyser LIAISON® XL (DiaSorin, Italy). HOMA-IR will be calculated from glucose and insulin. oxLDL will be analysed by solid phase two-site enzyme immunoassay (Mercodia Inc, Sweden).

Whole blood aggregation parameters, including amplitude, slope and lag time, will be assessed by a Chrono-Log Whole Blood Aggregometer, model 590 from Probe & Go (Endingen, Germany), which is based on the impedance method.

Thrombelastometry (TEM) parameters including coagulation time (CT), clot formation time (CFT), maximum clot firmness (MCF) and alpha angle will be assessed by the TEM coagulation analyser ROTEM®05 (Matel Medizintechnik, Austria). Prothrombin time (PT) and partial thromboplastin time (PTT) will be determined using the apparatus ACL Top LTS 300 (Werfen GmbH, Germany).

Malondialdehyde (MDA) will be determined by high-performance liquid chromatography (HPLC) and carbonyl proteins (CP) by a carbonyl protein enzyme-linked immunosorbent assay (ELISA) kit (Immundiagnostik AG, Germany). Redox state of albumin will be determined by separating albumin into three fractions; human mercaptalbumin (HMA), human non-mercaptalbumin1 (HNA1) and human non-mercaptalbumin2 (HNA2). HMA, HNA1 and HNA2 will be determined by means of HPLC.

The plasma levels of IL-5, IL-8, TNF-α, sTNFR1, sTNFR2, CCL2, CCL5, IL-1β and osteoprotegerin will be determined using a customised 9-Plex immunoassay (Human magnetic Luminex assay, R&D Systems, US).

Plasma levels of dp-ucMGP will be measured with the IDS-iSYS InaKtif MGP (dp-ucMGP) assay on an IDS-iSYS Multi-Discipline Automated System (ids Immunodiagnostic Systems, UK), according to the manufacturer’s instructions. Undercarboxylated osteocalcin will be determined by a sandwich ELISA kit (Novus Biologicals, Bio-Techne).

Plasma levels of BDNF and NGF will be determined using a customised multiplex immunoassay, which allows the simultaneous quantitation of multiple cytokines in a single sample (Human magnetic Luminex assay; R&D Systems).

For the measurement of mitochondrial DNA copy number (mtDNA-CN), DNA will initially be isolated from anticoagulated whole blood on a MagNA Pure instrument (Roche, Vienna, Austria). mtDNA-CN will subsequently be measured by multiplexed real-time quantitative polymerase chain reaction (qPCR) utilising ABI TaqMan chemistry (Applied Biosystems).

Tocopherols, retinol and β-carotene will be measured with ClinRep® kit (Recipe Chemicals + Instruments GmbH, Germany) by means of HPLC. Alpha-carotene, lutein, zeaxanthin, β-cryptoxanthin and lycopene will be determined and quantified via external standard curves. Vitamins C, K1 and K2 will also be determined by HPLC.

Total fatty acids (FAs), free and esterified, will be measured by the gas chromatograph–electron impact–mass spectrometer (GC-EI/MS), Agilent GC-MSD 5977 (Agilent, USA). Human plasma will be diluted in methyl-tert-butylether (MTBE) and methanol. Each sample will be spiked with a 400 nmol FA 17:0 as internal standard. The mass spectrometer will run in electron impact mode, and FAs are detected in full scan of m/z 80–400. Peak areas for FAs will be calculated by MassHunter and related to FA 17:0 internal standard peak areas. Quantities for FAs will be calculated by a one-point calibration (target FA vs FA 17:0).

#### Cognitive function

Cognitive function will be assessed via the Cognitive Functions Dementia index (CFD index), proposed by Jahn and Heßler [36]. Cognitive performance is estimated via the following 4 dimensions: (1) attention (alertness, shared attention, processing speed), (2) verbal long-term memory, (3) executive functions (spatial working memory, cognitive flexibility) and (4) expressive language (word fluency, object designation).

Participants will complete a series of tasks involving the above dimensions electronically and results of the individual tests, and a total score will be automatically calculated. Results are fed back by a percentage ranking via comparison to standard sample. Scores falling in a percentile rank between 16 and 100 are considered clinically normal, scores between percentile ranks 4 and 15 denote mild cognitive impairment, and those between percentile 0 and 3 denote severe cognitive impairment.

#### Quality of life

Physical and mental health-related quality of life will be assessed via completion of the Short Form Health Survey (SF-36). It consists of 36 questions assessing 8 domains, namely (1) physical functioning, (2) role limitations due to physical health, (3) role limitations due to emotional problems, (4) energy/fatigue, (5) emotional well-being, (6) social functioning, (7) pain and 8) general health. All items are scored on a scale from 0 to 100, with higher scores representing a better health state [37].

#### Stress profile

Participants’ stress profile will be assessed via completion of the Perceived Stress Scale (PSS-10) questionnaire developed by Cohen et al. [38]. It consists of 10 questions, and for each question, individuals are asked to select how often they felt a certain way in the last month, ranging from never to very often. Individual scores can range from 0 to 40 with higher scores indicating higher perceived stress. Depending on the score, individuals are classified as experiencing little, moderate or high levels of stress.

#### Physical activity assessment

To assess participants’ physical activity, the International Physical Activity Questionnaire—Short Form (IPAQ-SF) will be used. A previous study [39] has shown acceptable reliability (Spearman coefficient *r* = 0.54) for total activities, in German-speaking, middle-aged people, which is a similar cohort to ours. The IPAQ-SF comprises of 7 questions which ask completers about the frequency (days/ week) and duration (hours or minutes/ day) they spent being physically active in the last 7 days. The types of activities included are walking, moderate-intensity and vigorous-intensity activities. The calculations are based on the metabolic equivalents (MET) principle and the questionnaire suggests three levels of physical activity: low, moderate and high [40].

#### Upper respiratory tract symptoms

For an exploratory assessment of the incidence, duration and severity of upper respiratory tract symptoms, the Wisconsin Upper Respiratory tract Symptoms Survey (WURSS-21) will be used. The WURSS-21 questionnaire contains 21 questions and is an evaluative illness-specific quality of life instrument, designed to assess the negative impact of acute upper respiratory tract infection, presumed viral (the common cold) [41, 42].

#### Gastrointestinal symptoms

For an exploratory assessment of the incidence, duration and severity of gastrointestinal symptoms, the Gastro-Intestinal Symptoms Survey (GISS-16) will be used. The GISS-16 questionnaire was developed based on an adaptation of the WURSS-21. For the needs of this study, the survey includes 16 questions.

#### Nutritional assessment

Participants’ nutrition will be assessed via analysis of the food diaries recorded prior to the baseline visit, using the nutritional assessment program nut.s science database (version 1.32.81, Dato Denkwerkzeuge, Vienna, Austria). All participants will be provided with a copy of their nutritional output.

#### Data collection, storage and management

All team members will receive appropriate training on all measurements taken prior to the start of the trial. Collected data will be anonymised and linked to unique ID numbers, ensuring that participants’ confidentiality is maintained. Records that contain names or other personal identifiers will be stored separately from study records identified by ID number. Data collected in paper copies will be safely stored in locked cabinets. All study data will be recorded electronically on a server platform, where only authorised team members will have access to. In order to ensure accuracy of data, we will perform random data checks and range checks.

#### Sample size

The sample size calculation was performed according to omega-3 index changes as a key biomarker of cardiovascular health and TNF-α changes as a key biomarker of low-grade inflammation, following supplementation. Assuming a two-tailed t-test using 5% significance level (two-sided), a sample size of 18 and 19 subjects per group was determined to have 80% power to detect a 30% increase on omega-3 index [32] and a minimum decrease of 0.4 ng/L on TNF-α [43], respectively. Allowing for a dropout rate of 30% over 24 months, we aim to recruit 112 participants in total (*n* = 28 control group; *n* = 28 FVB group; *n* = 28 Omega group; *n* = 28 FVBO group).

### Statistical analysis

Statistical analysis will be performed using the Statistical Package for the Social Sciences (SPSS) software (version 24.0, IBM Corporation, Chicago, IL) by an independent statistician, blinded to participants’ group allocation. Metric data will be presented as mean ± SD. Statistical significance is set at *P* < 0.05. Normally distributed data fulfilling variance homogeneity will be analysed by one- and two-factorial (either ‘time’ or ‘time x treatment’) repeated measures analysis of variance (ANOVA) and covariance within each group and between all groups. Normal distribution will be determined by the Shapiro–Wilk test and homogeneity of variance by the Levene test. For post hoc analyses we will use the Bonferroni(–Holm) correction and/or Tukey’s post hoc test.

If it is not possible to use metric data, nonparametric tests will be used like the Friedman test (within group) and the Kruskal–Wallis test (between groups). If differences between groups reach significance, the Bonferroni–Holm method will be used to determine the localisation of the differences.

In addition, we will conduct comprehensive correlation analyses to compute relations within each outcome category and between the different outcome categories.

Statistical analyses will include data from all participants with an adherence to the study protocol (intervention compliance) of > 80%.

## Discussion

To the best of our knowledge, this is the first long-term intervention trial to assess the effect of separate as well as combined ingestions of an encapsulated juice powder concentrate and a plant-based omega fatty acid supplement on biomarkers of cardiovascular health, low-grade inflammation and indicators of ageing in middle-aged and elderly people. Considering that a large proportion of the population already struggles to meet the recommendations on fruit and vegetable and n3 PUFA intakes, whole food-based supplements might play a significant role to reach these targets.

Although the long-term intervention with the fruit and vegetable concentrate supplements and mixed omega fatty acid supplements used in this trial, over two years, has a novel approach and could provide interesting data, it could also have a drawback with regard to product compliance and participants’ commitment to follow-up visits. Nonetheless, we will implement a system of regular communication with the study’s participants in order to keep them motivated and engaged with the study. Additional limitations include the absence of a placebo group and the non-blinded design. However, keeping the study blinded over such a long period of time bears a high risk that participants find out the group they have been allocated to and the placebo group could lose motivation, imposing a compliance problem. We believe that the explorative character of this study, justifies the given design. Finally, although we ask participants to maintain their habitual diet during the entire duration of the study and we ask them to replicate their diet prior to each follow-up visit, there might be cases where participants change their habits considering the long duration of the study. Nevertheless, we do ask about their habits with the use of a questionnaire on every follow-up visit.

The advantages of our study are the comprehensive panel of biomarkers we intend to investigate, the observation period over 2 years, as well as the different outcome categories. Investigating supplements of plant-based origin in times where people are increasingly looking to incorporate plant-based alternatives in their diets compared to animal source products is of great importance, and relevance, specifically for the end user. In addition, previous studies investigating the supplements we intend to use have demonstrated not only the presence of a plethora of phytochemicals and the stability of omega fatty acids [29, 32], but also their absorption and bioavailability by the human body [32, 33, 44]. Since ageing is a process characterised by increased oxidative stress over time and chronic low-grade inflammation, it is of particular interest to investigate whether the active compounds present in the supplementation capsules could exert any health benefits and potentially slow down the process of ageing.

## Data Availability

We aim to publish results derived from this project, in international peer-reviewed scientific journals.

## Abbreviations

AA: Arachidonic acid
AI: Adequate intake
ALA: Alpha-linolenic acid
ApoA1: Apolipoprotein A1
BDNF: Brain-derived neurotropic factor
BMI: Body mass index
BP: Blood pressure
CCL2: Chemokine (C–C motif) ligand 2
CCL5: Chemokine (C–C motif) ligand 5
CFD index: Cognitive Functions Dementia index
CFT: Clot formation time
CONSORT: Consolidated Standards of Reporting Trials
CP: Carbonyl proteins
CRP: C-reactive protein
CT: Coagulation time
CVD: Cardiovascular disease
DHA: Docosahexaenoic acid
DPA: Docosapentaenoic acid
dp-ucMGP: Dephosphorylated-uncarboxylated Matrix GLA protein
EFSA: European Food Safety Authority
ELISA: Enzyme-linked immunosorbent assay
EPA: Eicosapentaenoic acid
FAs: Fatty acids
FVB: Fruit, Vegetable and Berry
FVBO: Fruit, Vegetable, Berry and Omega
GISS-16: Gastro-Intestinal Symptoms Survey-16
HbA1c: Haemoglobin A1c
HDL: High-density lipoprotein
HMA: Human mercaptalbumin
HNA1: Human non-mercaptalbumin1
HNA2: Human non-mercaptalbumin2
HOMA-IR: Homeostatic Model Assessment of Insulin Resistance
HPLC: High-performance liquid chromatography
hsCRP: High-sensitivity C-reactive protein
IL-1β: Interleukin 1-beta
IL-5: Interleukin-5
IL-8: Interleukin-8
IPAQ-SF: International Physical Activity Questionnaire-Short Form
LDL: Low-density lipoprotein
MCF: Maximum clot firmness
MDA: Malondialdehyde
MET: Metabolic equivalent
MTBE: Methyl-tert-butylether
mtDNA-CN: Mitochondrial DNA copy number
NGF: Nerve growth factor
OPG: Osteoprotegerin
oxLDL: Oxidised low-density lipoprotein
PSS-10: Perceived Stress Scale-10
PT: Prothrombin time
PTT: Partial thromboplastin time
qPCR: Quantitative polymerase chain reaction
RDA: Recommended daily allowance
SF-36: 36-Item Short Form Health Survey
SPIRIT: Standard Protocol Items: Recommendations for Interventional Trials
SPSS: Statistical Package for the Social Sciences
sTNFR1: Soluble tumour necrosis factor receptor 1
sTNFR2: Soluble tumour necrosis factor receptor 2
TEM: Thrombelastometry
TG: Triglycerides
TNF-α: Tumour necrosis factor alpha
uc-osteocalcin: Undercarboxylated osteocalcin
WURSS-21: Wisconsin Upper Respiratory tract Symptoms Survey-21

## References

[1] WHO, *Ageing and Health.* 2018.

[2] Calder PC (2017). Health relevance of the modification of low grade inflammation in ageing (inflammageing) and the role of nutrition. Ageing Res Rev.

[3] Yang T (2017). The impact of cerebrovascular aging on vascular cognitive impairment and dementia. Ageing Res Rev.

[4] Dauchet L (2006). Fruit and vegetable consumption and risk of coronary heart disease: a meta-analysis of cohort studies. J Nutr.

[5] Joshipura KJ (2001). The effect of fruit and vegetable intake on risk for coronary heart disease. Ann Intern Med.

[6] Hosseini B (2018). Effects of fruit and vegetable consumption on inflammatory biomarkers and immune cell populations: a systematic literature review and meta-analysis. Am J Clin Nutr.

[7] Macready AL (2014). Flavonoid-rich fruit and vegetables improve microvascular reactivity and inflammatory status in men at risk of cardiovascular disease—FLAVURS: a randomized controlled trial. Am J Clin Nutr.

[8] Root MM (2012). Combined fruit and vegetable intake is correlated with improved inflammatory and oxidant status from a cross-sectional study in a community setting. Nutrients.

[9] Biswas SK (2016). Does the interdependence between oxidative stress and inflammation explain the antioxidant paradox?. Oxid Med Cell Longev.

[10] Siti HN, Kamisah Y, Kamsiah J (2015). The role of oxidative stress, antioxidants and vascular inflammation in cardiovascular disease (a review). Vascul Pharmacol.

[11] Singh U, Devaraj S, Jialal I (2005). Vitamin E, oxidative stress, and inflammation. Annu Rev Nutr.

[12] Nooyens ACJ, Martin CR, Preedy VR (2015). Chapter 30 - Fruit and Vegetable Consumption and Cognitive Decline. Diet and nutrition in dementia and cognitive decline.

[13] Joseph JA, Shukitt-Hale B, Casadesus G (2005). Reversing the deleterious effects of aging on neuronal communication and behavior: beneficial properties of fruit polyphenolic compounds. Am J Clin Nutr.

[14] Del Gobbo LC (2016). ω-3 polyunsaturated fatty acid biomarkers and coronary heart disease: pooling project of 19 cohort studies. JAMA Intern Med.

[15] Harris WS, Del Gobbo L, Tintle NL (2017). The omega-3 index and relative risk for coronary heart disease mortality: estimation from 10 cohort studies. Atherosclerosis.

[16] Custodero C (2018). Evidence-based nutritional and pharmacological interventions targeting chronic low-grade inflammation in middle-age and older adults: a systematic review and meta-analysis. Ageing Res Rev.

[17] Bakker GC (2010). An antiinflammatory dietary mix modulates inflammation and oxidative and metabolic stress in overweight men: a nutrigenomics approach. Am J Clin Nutr.

[18] García-Calzón S (2015). Dietary inflammatory index and telomere length in subjects with a high cardiovascular disease risk from the PREDIMED-NAVARRA study: cross-sectional and longitudinal analyses over 5 y. Am J Clin Nutr.

[19] Senoner T, Dichtl W. Oxidative stress in cardiovascular diseases: still a therapeutic target? Nutrients, 2019;11(9). 10.3390/nu11092090 PMC6769522 31487802

[20] Petersson SD, Philippou E (2016). Mediterranean diet, cognitive function, and dementia: a systematic review of the evidence. Adv Nutr.

[21] van den Brink AC (2019). The Mediterranean, Dietary Approaches to Stop Hypertension (DASH), and Mediterranean-DASH Intervention for Neurodegenerative Delay (MIND) diets are associated with less cognitive decline and a lower risk of Alzheimer's disease: a review. Adv Nutr.

[22] Barbaresko J (2013). Dietary pattern analysis and biomarkers of low-grade inflammation: a systematic literature review. Nutr Rev.

[23] Petra Rust, V.H., Jürgen König. Österreichischer Ernährungsbericht 2017. 2017; Available from: https://broschuerenservice.sozialministerium.at/Home/Download?publicationId=528.

[24] Austria S. Österreichische Gesundheitsbefragung 2019;2019.

[25] Azzolina, D., et al. Nutrients and caloric intake associated with fruits, vegetables, and legumes in the elderly european population*.* Nutrients2020;**12**(9). 10.3390/nu12092746 PMC7551243 32916924

[26] Richter CK (2019). n-3 Docosapentaenoic acid intake and relationship with plasma long-chain n-3 fatty acid concentrations in the United States: NHANES 2003–2014. Lipids.

[27] Jin Y (2010). Systemic inflammatory load in humans is suppressed by consumption of two formulations of dried, encapsulated juice concentrate. Mol Nutr Food Res.

[28] Lamprecht M (2013). Supplementation with a juice powder concentrate and exercise decrease oxidation and inflammation, and improve the microcirculation in obese women: randomised controlled trial data. Br J Nutr.

[29] Bresciani L (2015). (Poly)phenolic characterization of three food supplements containing 36 different fruits, vegetables and berries. PharmaNutrition.

[30] German Society for Nutrition, A.S.f.N., Swiss Society for Nutrition, Reference values for nutrient intake. 2019.

[31] EFSA Panel on Dietetic Products, N., and Allergies (NDA), Scientific opinion on dietary reference values for fats, including saturated fatty acids, polyunsaturated fatty acids, monounsaturated fatty acids, trans fatty acids, and cholesterol*.* EFSA J. 2010; 8(3):1461.

[32] Dams S (2020). Effects of a plant-based fatty acid supplement and a powdered fruit, vegetable and berry juice concentrate on omega-3-indices and serum micronutrient concentrations in healthy subjects. Int J Food Sci Nutr.

[33] Dams, S., et al. An encapsulated fruit, vegetable and berry juice powder concentrate increases plasma values of specific carotenoids and vitamins. Int J Vitam Nutr Res. 2019;1–10. 10.1024/0300-9831/a000609 31726948

[34] Bäckman L (2005). Cognitive impairment in preclinical Alzheimer's disease: a meta-analysis. Neuropsychology.

[35] Chan AW (2013). SPIRIT 2013 explanation and elaboration: guidance for protocols of clinical trials. BMJ.

[36] Jahn TH, J.B. Cognitive functions dementia: manual, S. GmbH, Editor. 2017: Mödling.

[37] Ware JE, Jr, Sherbourne CD. The MOS 36-item short-form health survey (SF-36). I. Conceptual framework and item selection. Med Care, 1992;30(6):473–83. 1593914

[38] Cohen S, Kamarck T, Mermelstein R (1983). A global measure of perceived stress. J Health Soc Behav.

[39] Mäder U (2006). Validity of four short physical activity questionnaires in middle-aged persons. Med Sci Sports Exerc.

[40] Craig CL (2003). International physical activity questionnaire: 12-country reliability and validity. Med Sci Sports Exerc.

[41] Barrett B (2009). Validation of a short form Wisconsin Upper Respiratory Symptom Survey (WURSS-21). Health Qual Life Outcomes.

[42] Brown RL, Obasi CN, Barrett B. Rasch analysis of the WURSS-21 dimensional validation and assessment of invariance. J Lung Pulm Respir Res. 2016;3(2). 10.15406/jlprr.2015.03.00076 PMC5089813 27812536

[43] Lamprecht M (2007). Several indicators of oxidative stress, immunity, and illness improved in trained men consuming an encapsulated juice powder concentrate for 28 weeks. J Nutr.

[44] Bresciani L, et al. Absorption profile of (poly)phenolic compounds after consumption of three food supplements containing 36 different fruits, vegetables, and berries. Nutrients, 2017;9(3). 10.3390/nu9030194 PMC5372857 28245627

